# Involvement of Connexin Hemichannels in the Inflammatory Response of Chronic Diseases

**DOI:** 10.3390/ijms19092469

**Published:** 2018-08-21

**Authors:** Juan C. Sáez, Colin Green

**Affiliations:** 1 Departamento de Fisiología, Facultad de Ciencias Biológicas, Pontificia Universidad Católica de Chile, Alameda 340, 8331150 Santiago, Chile; 2 Instituto de Neurociencias, Centro Interdisciplinario de Neurociencias de Valparaíso, Universidad de Valparaíso, 2381850 Valparaíso, Chile; 3 Department of Ophthalmology, New Zealand National Eye Centre, University of Auckland, Auckland 1142, New Zealand

Over the last decade it has become evident that under normal conditions connexin hemichannels are either not expressed (e.g., skeletal muscle) or are expressed in very low numbers with low open probability in various mammalian tissues (e.g., liver and central nervous system (CNS)). However, both the number and activity of these non-selective channels are drastically increased in different cell types under pathological conditions. Since the inflammatory response is a consequence and/or the cause of most diseases, connexin hemichannels have been implicated as a common factor in numerous chronic pathological indications (including muscular dystrophy, amyotrophic lateral sclerosis, ongoing effects of CNS trauma, stroke or ischemia, glaucoma, diabetic retinopathy and macular degeneration, Alzheimer’s and Parkinson’s disease, epilepsy, chronic pain, brain and other tumors, and infectious diseases). However, further work is needed to fully understand hemichannel regulatory mechanisms and their involvement in the outcome of the different disease conditions.

Currently, the molecular target of different clinically available anti-inflammatory agents block one (or at most two) intracellular inflammatory signaling pathways, leaving several others active, and which persist despite chronic treatment, and alter the phenotype of inflamed cells. The phenotypic changes explain why cells stop performing their normal functions and subsequently lead to organ dysfunction. Consequently, the chronic use of many available anti-inflammatory compounds can result in kidney, liver, or heart dysfunction, amongst other complications. Connexin hemichannel regulation offers an alternative approach. Connexin hemichannels have been shown to be permeable to calcium ions and ATP which activate intracellular inflammatory signaling pathways. Therefore, selective inhibitors of connexin hemichannels are expected to be useful for the long-term treatment of inflammatory disease. These agents may provide an entire new generation of anti-inflammatory pharmaceuticals. Indeed, it is important to emphasize the evidence for connexin hemichannels as new molecular targets in order to prevent the activation of most intracellular inflammatory pathways.

This issue of the *International Journal of Molecular Sciences* focuses on the involvement of connexin hemichannels in the inflammatory response, emphasising chronic pathological conditions. It brings together substantial evidence for connexin hemichannel-mediated tissue dysfunction associated with a diverse range of chronic disease indications, thus supporting the translation of hemichannel regulation into clinical application.

For instance, Valdebenito and co-workers [1] have provided novel information revealing the importance of a cell–cell communication pathways mediated by connexin-based channels in cancer, including gap junction channels, hemichannels, and tunneling nanotubes. Artificial intelligence and machine learning are proposed as new approaches that could provide insights into the intercellular transfer of cell signals. They also proposed that the identification of these cell–cell communication systems and their associated signaling might identify new targets to prevent or reduce the effects of cancer. In addition, Rhett and Yeh [2] have reviewed the possible involvement of hemichannels in the inflammatory response associated with the progression of breast cancer. They have also proposed connexin-based therapeutics to reduce the inflammatory component in that disease. In contrast to hemichannels, Uzu and collaborators [3] have discussed whether gap junctional communication could be cancer promoting or suppressing, depending on what permeates through the gap junction channels. However, they have also stressed the need to focus on the possible role of connexin proteins and hemichannels in the cells of brain tumors. In the nervous system under inflammatory conditions, Lagos Cabré and collaborators [4] have shown that reactive astrocytes acquire a migratory phenotype. They have demonstrated an important role for connexin hemichannels in Thy-1-induced astrocyte migration.

The role of connexin hemichannels in inflammatory responses within peripheral tissues such as retina, heart, liver, and kidney are also described. Mugisho and collaborators [5] have analyzed the expression of connexin43 in vitro using a mouse model of diabetic retinopathy as well as in human donor tissues. They found a reduced expression of connexin43 with diabetes, but hyperglycemia and inflammation present in diabetic retinopathy appear to increase connexin43 expression, suggesting a causal role of connexin43 channels in the progression of that disease. Similarly, Gómez and collaborators [6] have shown that angiotensin II applied to MES-13 cells—a cell line derived from mesangial cells—induces oxidative stress and the generation of pro-inflammatory cytokines, both of which are associated with increased membrane permeability mediated by non-selective membrane channels, including connexin43 hemichannels. They propose that angiotensin II-induced mesangial cell damage could be effectively inhibited by simultaneous blockade of the RhoA/ROCK-dependent pathway and connexin43 hemichannels, which could be relevant for the prevention of chronic kidney dysfunction. Another chronic dysfunction alleviated by the inhibition of connexin43 hemichannels is liver fibrosis. Crespo Yanguas et al. [7] have demonstrated that treatment with the hemichannel blocking peptide TAT-Gap19 alleviates liver fibrosis induced by the administration of thioacetamide to Balb/c mice. The administration of TAT-Gap19 lowered the degree of fibrosis that accompanies superoxide dismutase over activation, and reduced the production of inflammatory cytokines. These findings again reveal the therapeutic potential of connexin43 hemichannel blockers in the treatment of chronic disease, in their case liver fibrosis. Moreover, Johnson and Camelliti [8] have reviewed the possible involvement of connexin-based channels in non-myocyte cells of the heart, and propose that they contribute to arrhythmias and adverse ventricular remodeling following myocardial infarction, and are associated with the initiation and development of atherosclerosis. Consequently, they propose that interventions targeting connexins are avenues with great potential. Further supporting the role of intracellular Ca^2+^ and reactive oxygen species (ROS) on cell damage, Pecoraro et al. [9] have focused on doxorubicin, a potent antineoplastic agent that is unfortunately also cardiotoxic, limiting its use in cancer therapy. Doxorubicin was found to impair Ca^2+^ homeostasis, but also to enhance the amount of connexin43 expression in addition to affecting its localization. Keeping in mind that intracellular Ca^2+^ and ROS activate connexin43 hemichannels, which are permeable to Ca^2+^ and ROS, it is tempting to propose that connexin43 hemichannel blockers might reduce the cardiotoxicity of doxorubicin. Notably, the same group [10] demonstrated in a second contribution that diazoxide, a specific opener of mitochondrial KATP channels that is widely used for its cardioprotective effects, also increases the amount of connexin43 expressed. This finding now needs follow-up with functional assays of gap junctional communication and hemichannel activity in the sarcolemma and in mitochondrial membranes, but again opens a possible avenue to regulate the functional state of connexin-based channels in inflammatory responses.

Clearly, more studies are required to fully understand how a reduction in gap junctional communication but, conversely, a gain in plasma membrane hemichannel activity, as well as possible hemichannel activity in mitochondria and channel-independent functions of connexins, may impact chronic disease. This special edition marks a significant step in the right direction.
